# A Review on Biopolymer-Based Biodegradable Film for Food Packaging: Trends over the Last Decade and Future Research

**DOI:** 10.3390/polym15132781

**Published:** 2023-06-22

**Authors:** Andi Dirpan, Andi Fadiah Ainani, Muspirah Djalal

**Affiliations:** 1 Department of Agricultural Technology, Faculty of Agriculture, Hasanuddin University, Makassar 90245, Indonesia; 2 Center of Excellence in Science and Technology on Food Product Diversification, Makassar 90245, Indonesia; 3 Research Group for Post-Harvest Technology and Biotechnology, Makassar 90245, Indonesia

**Keywords:** active packaging, polysaccharides, proteins, lipids, bibliometric analysis

## Abstract

In recent years, much attention has been paid to the use of biopolymers as food packaging materials due to their important characteristics and properties. These include non-toxicity, ease of availability, biocompatibility, and biodegradability, indicating their potential as an alternative to conventional plastic packaging that has long been under environmental scrutiny. Given the current focus on sustainable development, it is imperative to develop studies on biopolymers as eco-friendly and sustainable food packaging materials. Therefore, the aim of this review is to explore trends and characteristics of biopolymer-based biodegradable films for food packaging, analyze the contribution of various journals and cooperation between countries, highlight the most influential authors and articles, and provide an overview of the social, environmental, and economic aspects of biodegradable films for food packaging. To achieve this goal, a bibliometric analysis and systematic review based on the PRISMA method were conducted. Relevant articles were carefully selected from the Scopus database. A bibliometric analysis was also conducted to discuss holistically, comprehensively, and objectively biodegradable films for food packaging. An increasing interest was found in this study, especially in the last 3 years with Brazil and China leading the number of papers on biodegradable films for food packaging, which were responsible for 20.4% and 12.5% of the published papers, respectively. The results of the keyword analysis based on the period revealed that the addition of bioactive compounds into packaging films is very promising because it can increase the quality and safety of packaged food. These results reveal that biodegradable films demonstrate a positive and promising trend as food packaging materials that are environmentally friendly and promote sustainability.

## 1. Introduction

The use of plastics in food packaging is widespread globally because of desirable characteristics such as being lightweight and cost-effective, having good mechanical properties, and being easy to manufacture [1]. However, the high dependence on plastics, especially those made from fossil fuels, is extremely harmful to the environment [2]. In addition, it affects the environment on both land and ocean and the health of living beings [3]. Plastics also play significant roles in global greenhouse gas (GHG) emissions. In 2019, plastics were responsible for 1.8 billion tons of GHG emissions, which accounted for approximately 3.4% of total global emissions [4]. Most of these emissions (90%) resulted from the production and conversion of plastics from fossil fuels [4]. Furthermore, by 2060, GHG emissions from the entire life cycle of plastics will more than double, reaching 4.3 billion tons [5]. A recent study Sandhu et al. [6] found that approximately 200 million tons of synthetic plastics are produced annually, which consumes a large amount of non-renewable petroleum resources, emits hundreds of millions of tons of carbon dioxide (CO_2_), and results in the production of toxic materials that are harmful to human health. Kumar et al. [3] also reported that in 2019, the global production of plastic was at 370 million tons, and only a fraction of the plastic produced could be recycled (9%), with 12% burned through combustion and the rest left in the environment or disposed of in landfills. The end-of-life impact of plastics refers to the stage in the life cycle of plastic products where they reach the end of their useful life and are then disposed of or recycled [7]. The worst situation regarding the use of conventional plastics entering the environment is the length of time it takes to decompose, which can lead to negative impacts such as environmental pollution, greenhouse gas emissions, climate change, and ecosystem damage [3,8]. One solution to overcome this problem is through the development and use of biodegradable plastics that are environmentally friendly and harmless to health. Biodegradable materials offer a viable option for food packaging, and once they reach the end of their useful life, they can be composted to recover their carbon atoms, which will enrich the soil and allow the creation of new foods or materials [9,10,11].

Recent technological progress has facilitated the production of biodegradable films through the utilization of materials sourced from renewable and environmentally friendly origins. These advancements involve employing natural biopolymers, blending different biopolymers to enhance mechanical characteristics while preserving biodegradability, and creating nanocomposite films to improve film stability [12,13]. Biodegradable films are made from biobased polymers. Biobased polymers are plastics made from renewable resources such as those sourced from natural biopolymers (e.g., polysaccharides, proteins, and lipids), synthetic biopolymers (e.g., polylactic acid and polybutylene succinate), or microbial biopolymers (e.g., polyhydroxy alkanoates (PHA) and bacterial cellulose) [14,15].

Conventional plastics based on fossils are currently associated with concerns about resource depletion, unstable prices and greenhouse gas emissions [16]. So, there is a need to explore alternative natural resources that are renewable and have a low carbon footprint. As mentioned earlier, traditional plastics are manufactured using fossil fuels such as petroleum and natural gas, whereas biodegradable plastics are derived from renewable sources such as plant and microbial biomass. These renewable biopolymers possess chemical and mechanical properties that are comparable to those of conventional plastics [16,17]. The use of renewable raw materials is expected to be able to shift dependence from non-renewable fossil fuels. Biodegradable films have the advantage of being decomposed into natural components through biological processes [18]. Biodegradable plastics typically undergo complete biodegradation within a span of 3–6 months, whereas conventional plastics require several centuries to break down [19]. This faster breakdown process of biodegradable plastics enables them to return to the environment more quickly and with fewer harmful residues than conventional plastics [17]. Consequently, this helps mitigate the long-term buildup of waste in the environment. According to the findings of the Intergovernmental Panel on Climate Change (IPCC), the use of biopolymers has the potential to play a major role in global warming mitigation efforts of up to 1.5 degrees Celsius, which is expected to help eliminate up to 20% of carbon dioxide (CO_2_) [20].

Recent research trends show promising growth in the utilization of natural biopolymers as part of a long-term global sustainable development strategy [21]. However, it should be noted that biopolymers generally exhibit lower mechanical and chemical resistance compared to conventional petroleum-based plastics [22]. One potential solution to improve the properties of biopolymers is the use of polymer blends, which can reduce the repulsive forces in the biopolymer chain [23]. Food waste has also been widely utilized for the production of biodegradable plastics. Ranganathan et al. [24] reviewed various food wastes used for the production of biodegradable packaging, including waste from potato processing, fish waste, fruit and vegetable waste, poultry waste, etc. Then, thermoplastic starch and soybean pulp derived from soybean waste can be used in the production of disposable goods and packaging materials [25]. In addition, the mixture of polysaccharides and okra plant waste also successfully became a value-added biopolymer that can be used for food packaging applications [26]. Thus, the production of biopolymers from food waste can serve as an important step in reducing the disposal of food waste in landfills and waterways. By reducing waste and pollution, these films can help ensure that the Earth remains a healthy and livable place for future generations.

There is sufficient literature available on the use and development of biopolymer-based biodegradable films for food packaging. However, reviews on bibliometric analysis that comprehensively examine the trends and development patterns of biodegradable films for food packaging are scarce. Thus, this review intends to fill the knowledge gap in this study. This review conducted a bibliometric analysis using statistical and quantitative methods to collect published articles. The bibliometric analysis uses statistical and graphical techniques to identify scientific progress in several aspects of the field by assessing and displaying research patterns and organizing bibliographic data into a single document. By analyzing the relevant scientific literature, researchers can gain a comprehensive understanding of the current state of the field, identify gaps and challenges in knowledge, and direct future research efforts [27,28]. Therefore, this study aimed to conduct a literature review and bibliometric analysis of biodegradable films for food packaging to reveal the most commonly used biodegradable materials, level of research activity, and geographical distribution of research, applications, type and duration of biodegradation, and an overview of the social, environmental, and economic aspects of biodegradable films for food packaging. In addition, by analyzing references of relevant articles, this bibliometric analysis can determine the most influential articles and researchers in the field and direct future research efforts. With this aim, it is hoped to enlighten the readers about the extent of research and progress that has been made in this field in the last decades. In addition, the intended audience for this study consists of professionals and researchers working in the fields of food technology and industrial engineering, who are actively seeking technically sound, economical and sustainable innovative technologies for the food packaging industry.

## 2. Materials and Methods

The mixed-methods approach used in this literature review includes both a bibliometric analysis and a systematic reviews [29,30]. This method combines quantitative and qualitative analyses and used numeric data and explanations [29]. The use of a blended approach provides a comprehensive perspective and enables theory building and empirical evaluation.

### 2.1. Data Sources

**Figure 1:**
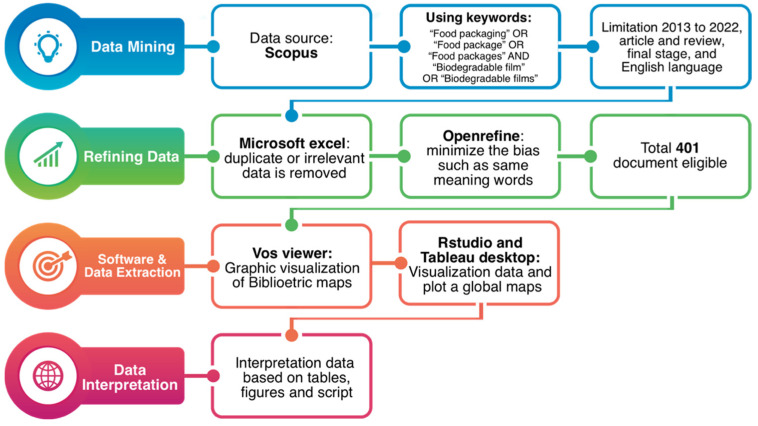
Flowchart of the bibliometric analysis.

Data for this study were extracted from Scopus (scopus.com), which is a renowned research database. To minimize bias and maintain consistency amidst database changes, data collection was conducted on a single day, 21 December 2022. The string search methodology uses quotation marks, and Boolean operators (“AND” and “OR”) are used. The search keywords used are TITLE-ABS-KEY “Food packaging” OR “Food packages” OR “Food packages” AND “Biodegradable films” OR “Biodegradable film”. The selected documents met the following criteria: published between 2013 and 2022, written in English, article and review document type, and in the final publication stage. Any documents failing to meet these criteria were excluded. Data were downloaded in CSV format and organized in Microsoft Excel for easy data management, with duplicate data removed. Then, the data are inputted into Openrefine. The Openrefine version 3.6.1 application (https://openrefine.org (accessed on 22 December 2022)) was used to combine words that have the same meaning: for example, “food package and food packaging” and “antioxidant activity and antioxidant activities”. In addition, singular and plural forms of the same word, e.g., active films and active film and biopolymer and biopolymers, were also combined. Ultimately, a total of 401 documents were used for bibliometric analysis. Figure 1 provides an overview of the bibliometric analysis process through a flowchart.

### 2.2. Data Analysis

The articles obtained from the literature search underwent a bibliometric analysis to gather metrics on publication details, country, journal sources, keywords, and other relevant parameters. The analysis was conducted utilizing functions in RStudio, Tableau, and Vosviewer software. Rstudio version 4.2.1 was used to visualize data three-field plots. Tableau version 2022.2.1 (https://www.tableau.com (accessed on 22 December 2022)) was used to visualize the analyzed data. VOSviewer software version 1.6.15.0 (https://www.vosviewer.com (accessed on 22 December 2022)) was used to create maps based on the network data. The size of the circle was determined by the weight of the item, i.e., the greater the weight, the larger the circle and the source, whereas the color determines the cluster that the item belongs to, the lines between the circles represent links, and the distance between the circles indicates the strength of the relationship between the analyzed terms [31].

Following the bibliometric analysis, a systematic evaluation of articles was conducted. Initially, the titles and abstracts of the search results were examined based on the predetermined eligibility criteria. Subsequently, the full articles of the papers selected during the title/abstract screening phase were evaluated to ensure compliance with the eligibility criteria. The criteria used to determine the eligibility of relevant articles were established in an impartial and independent manner.

- Inclusion criteria: Study period between 2013 and 2022; studies in the final publication phase; publications in the English language; document types: article and review
- Exclusion criteria: Publications in languages other than English; theses, dissertations, books, book chapter, and conference papers; and gray literature.

**Figure 2:**
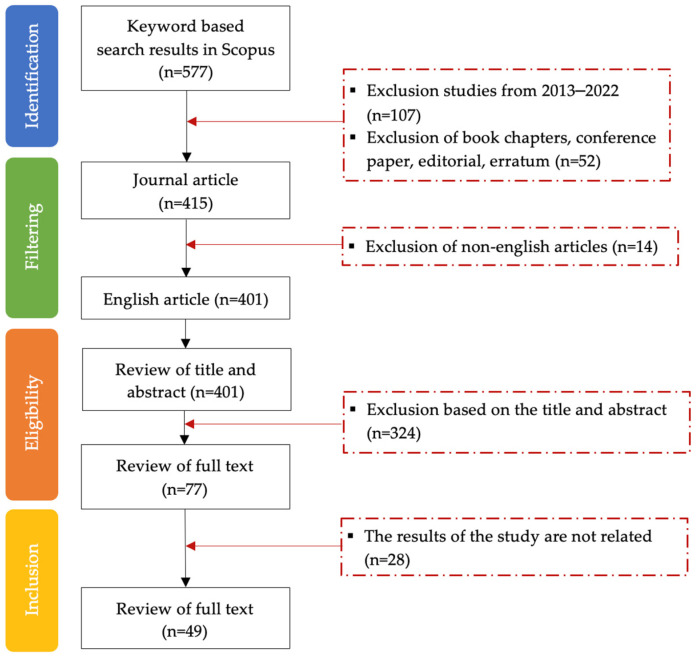
The preferred reporting items for systematic reviews, following the PRISMA methodology.

A flow diagram illustrating the article selection process for the systematic review, following the PRISMA methodology [32,33], is presented in Figure 2.

## 3. Overview of Biodegradable Polymers

**Figure 3:**
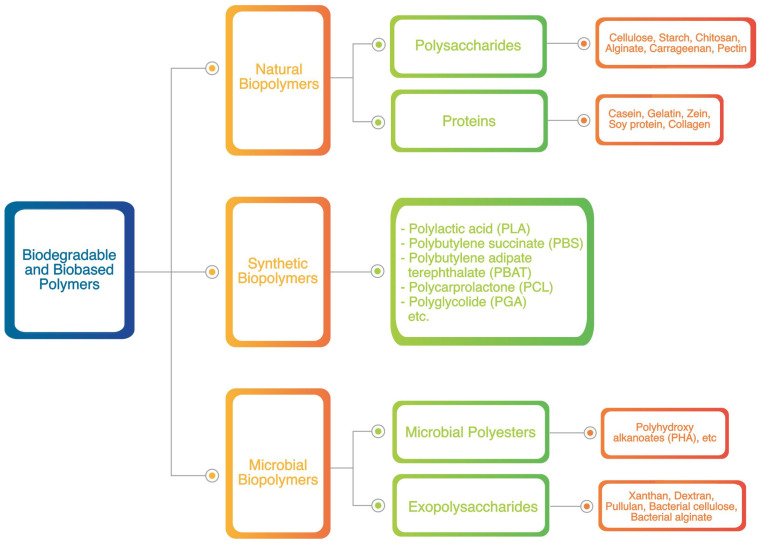
Classification of biodegradable polymers.

Recently, replacing non-degradable plastic packaging with biodegradable packaging materials has become more common. In the environment, biodegradable polymers can be broken down under the right environmental conditions such as humidity, temperature, oxygen availability, and presence of living creatures. This process leaves no harmful substances behind and does not negatively affect the environment. Biopolymer-based packaging materials are being investigated as an alternative to conventional plastics because of their biocompatibility, safety, and rate of biodegradation [34]. Biodegradable and biobased polymers based on their source materials are classified in three forms, including natural, synthetic, and microbial biopolymers, which are presented in detail in Figure 3. In the manufacturing of biobased polymers, the materials such as starch, organic acids, and synthetic carbonyl are added to make them biodegradable in the environment [35].

### 3.1. Natural Biopolymers

Generally, natural biopolymers are extracted naturally from polysaccharides, lipids, and proteins. Polysaccharides may consist of cellulose, alginate, starch, chitosan (CS), pectin, gum, carrageenan, pullulan, or derivatives of these substances. However, these materials have drawbacks when applied in biopolymers production. Biopolymer-based materials such as starch and cellulose have poor water vapor barrier characteristics because of their hydrophilic nature. These attributes contribute to a reduction in the mechanical strength of biopolymer films and impede their long-term stability, making them susceptible to moisture content [36,37,38]. Poor film processability, brittleness, and susceptibility to breakage are some other drawbacks. In general, protein-based films have excellent mechanical characteristics and are effective barriers to oxygen gas at moderate relative humidity. However, their hydrophilic nature made their water vapor barrier quality not very effective [39]. According to their origin, proteins can be grouped into two categories: those of plant origin, such as wheat gluten, soy, and corn zein, and those of animal origin, such as collagen, whey, casein, and gelatin. Casting is a frequently used method in the production of films composed of polysaccharides and proteins [15]. Two main stages are involved in the production of films using the casting method [40]: (1) dispersion or dissolution of the biodegradable polymer into a suitable solvent and (2) vaporization of the biopolymer in an environment under regulated conditions.

### 3.2. Synthetic Biopolymers

Synthetic biopolymers are generally produced through chemical processes from biological monomers. These materials include aliphatic–aromatic copolymers, aliphatic polyesters, polylactides, aliphatic copolymers (CPLA), and polylactides, and they are made from renewable biobased monomers such as poly lactic acid (PLA) and oil-based monomers such as polycaprolactone [41]. PLA is a biopolyester polymerized from lactic acid monomers, which is an excellent example of a polymer that can be made by chemical synthesis using renewable biobased monomers. Moreover, PLA is one of the most promising biobased polymers because of its availability and proven recyclability, compostability, and potential to replace conventional plastic materials. However, the use of PLA for food packaging is currently restricted because of its poor mechanical and barrier qualities. Adjusting the chemical composition of PLA and changing its molecular features made it possible to create and balance material qualities. Therefore, most studies have focused on improving the characteristics of PLA by adding nanoparticles and plasticizers [42,43,44].

### 3.3. Microbial Biopolymers

Microbial biopolymer consists of polymers produced by microorganisms or bacteria that have undergone genetic modification. PHA is one such polyester synthesized through the bacterial fermentation of sugars and lipids. PHA polyester is biodegradable and biocompatible and can be obtained from renewable resources [41]. PHA polymers generally exhibit good UV resistance and are insoluble in water, relatively resistant to hydrolytic damage, soluble in chloroform and chlorinated hydrocarbons, and non-toxic; however, they have a low resistance to acids and bases [45]. PHAs are potential materials that can compete with conventional plastics made from fossil fuels in the food packaging industry because of their hydrophobic properties and flexibility in mechanical properties [35].

## 4. Results and Discussion

### 4.1. Bibliometric Analysis and Scientific Performance

**Figure 4:**
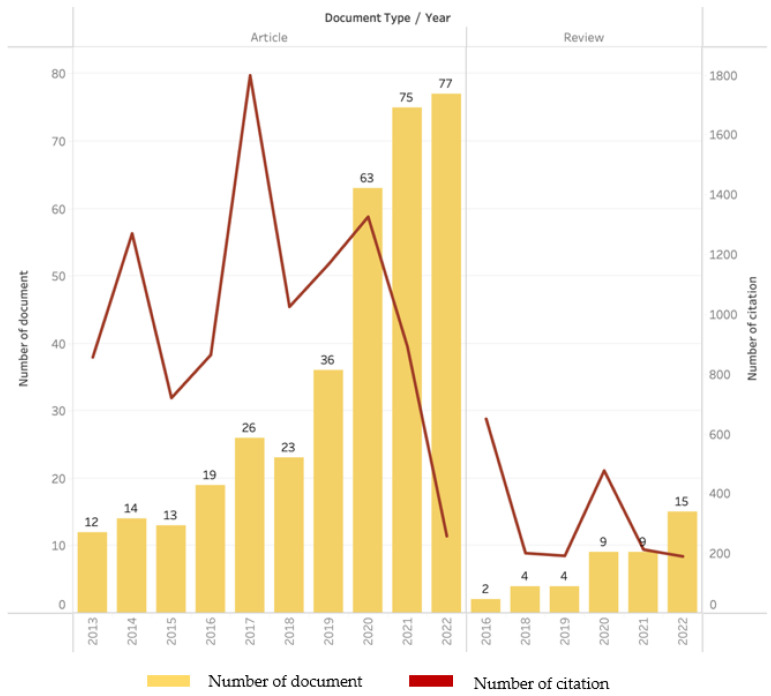
Number of publications and citations on biodegradable films for food packaging application.

Based on the results of the bibliometric analysis, studies on biodegradable film-based food packaging films show a trend that tends to increase annually. The development of this study can be seen from two types of documents, i.e., articles and reviews, based on the number of documents published and number of citations annually (Figure 4). From 2013 to 2022, the number of documents published in this study was 401, providing details of 358 articles (89.28% of the total documents) and 43 reviews (10.72% of the total documents) with a growth rate of 25.4% annually. In 2019, studies on this theme and discussed by researchers peaked in 2022 with 77 articles and 15 reviews. In addition, the highest annual numbers of document citations for articles and reviews were in 2017 and 2016: 1799 and 650, respectively. Numerous documents and citations are based on the increasing public awareness of environmental issues and better sustainability by reducing the use of conventional (nonbiodegradable) packaging, and this suggests that the use of environmentally friendly food packaging is gaining traction these days and will continue to increase. This is consistent with the findings of Sani et al. [14] and Nygaar et al. [35], who revealed that the interest in developing biodegradable packaging films to replace non-biodegradable plastic packaging materials has recently attracted growing attention.

### 4.2. Bibliometric Analysis of Country Performance

**Figure 5:**
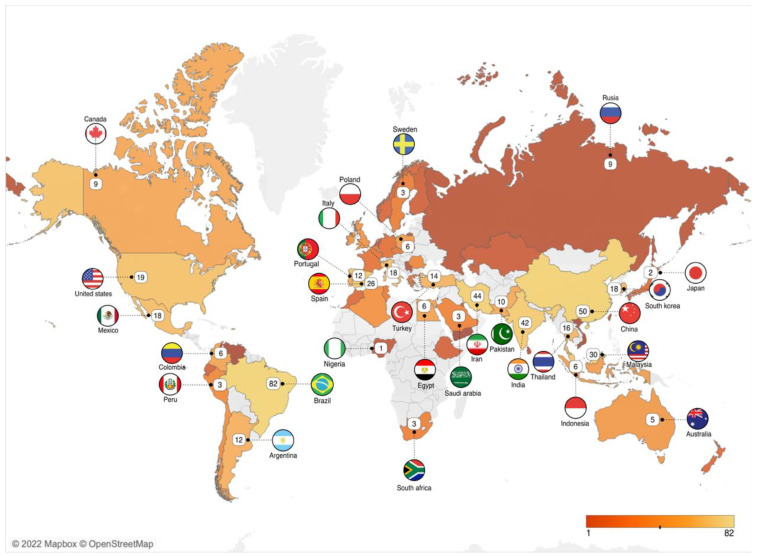
Representative map of scientific publications on the study of biodegradable films for food packaging production per country between 2013 and 2022.

Figure 5 shows the distribution of authors based on their nationality. Overall, 57 countries contributed to the study of biodegradable films for food packaging, which spread across five continents, i.e., South America, North America, Africa, Asia, Europe, and Australia. Brazil has the most document production (*n* = 82) followed by China (*n* = 50), Iran (*n* = 44), India (*n* = 42), and Malaysia (*n* = 30). Brazil has a high number of scientific publications because it is one of the largest plastic producers in the world. Brazil is among the top five countries as the largest producer of biodegradable plastics, along with China, the United States, Germany, and Canada [46]. Moreover, the availability of cheap raw materials is likely to bode well for the market in South America (Brazil) [47]. According to GLobalData, the market of biodegradable plastics in Brazil was worth $375 million in 2021, with a market volume of 105,000 tons. From 2021 to 2026, the global market for biodegradable plastics is expected to continuously expand at a compound annual growth rate of >24%.

In addition, the countries were also ranked based on the number of citations (Table 1). Iran has the highest number of citations (*n* = 2234 citations) followed by Spain (*n* = 2044) and Brazil (*n* = 1422). Iran also has high levels of plastic waste production and usage. Thus, to reduce the potential effect on the natural environment, the United Nations in Iran suggested replacing the use of disposable plastic packaging with environmentally friendly packaging [48]. This has prompted Iran to actively conduct various studies related to biodegradable packaging.

### 4.3. Bibliometric Analysis of the Most Relationship between the Journals, Countries, and Keywords

**Figure 6:**
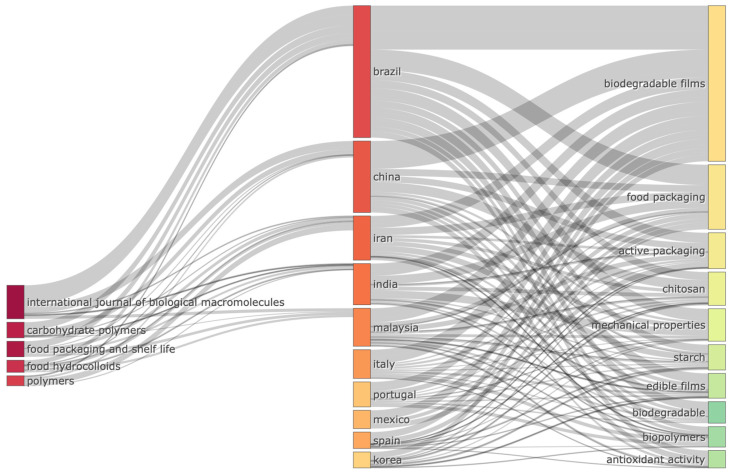
Three-field plot of related sources (journal), countries, and keywords on biodegradable film packaging.

Figure 6 presents the relationship between the journals that published the most documents, most productive countries, and keywords frequently used in the study of biodegradable films for food packaging. As shown in Figure 6, documents published in the International Journal of Biological Macromolecules are mostly written by Brazilian researchers and focus on several keywords regarding biodegradable films and food packaging. According to Matheus et al. [49], Brazil has led research on the development of starch-based films. Moreover, Brazilian researchers have successfully produced CS-based biodegradable films [50,51,52,53] from gelatin [54] and starch [55,56,57,58].

### 4.4. Analysis of Keywords

**Figure 7:**
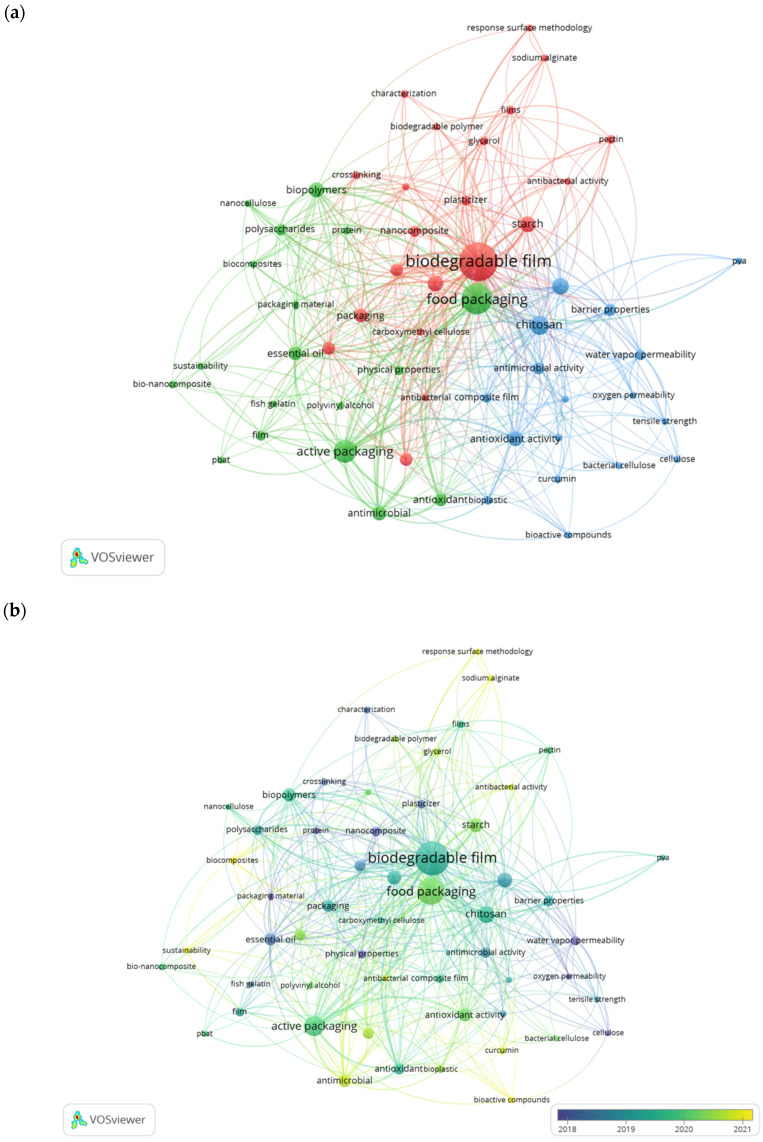
(**a**) Clusters of keywords in publications on the biodegradable film for food packaging; (**b**) Evolution of the said keywords through the period 2018 to 2021.

Keyword analysis can show current trends and thus track the development of scientific research [59]. The co-occurrence of the most popular author keywords was visualized using VOSviewer (Figure 7a). The minimum keyword occurrence was set at five times; out of the total of 940 keywords, 56 were found to fulfill the analysis. Each circle represents a keyword, and the size of the circle represents the number of publications that have the corresponding term in the keyword list. The color of the circle indicates the relationship between keywords and being in the same cluster [60]. Figure 7a shows the distribution of keywords according to their occurrence. The five most common keywords are biodegradable film (148), food packaging (93), active packaging (53), CS (38), and antimicrobial (20). From the results, three clusters were obtained, which were distinguished as red, yellow, and blue for the first, second, and third clusters, respectively (Figure 7a). In addition, the analysis of the significance of keywords over a period, e.g., 2018–2021, can see the development of research trends related to each of the main issues. Figure 7b presents this information in the form of a bibliometric map. In this study, the progression of keywords is shown in purple to yellow, which provides valuable information about current trends.

In Figure 7a, the first cluster (red) focuses on biodegradable film as environmentally friendly packaging, and biodegradable film (148 occurrences) is the most prominent keyword in this cluster. Polysaccharides derived from natural polymers dominate in biodegradable films including starch (56 occurrences), gelatin (32 occurrences), pectin (8 occurrences), and sodium alginate (5 occurrences). Polysaccharide-based packaging materials are generally relatively cheap and abundantly available and have some special features in their function as packaging materials. Although they are often hydrophilic and have poor water-vapor barrier qualities, polysaccharide-based biodegradable films are efficient barriers for the transfer of gases such as O_2_ and CO_2_. The gas barrier properties of polysaccharide-based films are also essential in preventing solvent penetration in food and slowing down the loss of organic vapors (aroma compounds) during storage, which can result in toxicity or quality deterioration.

The second cluster (green) focuses on food packaging (93 occurrences). At present, food packaging is often associated with the addition of dual functions, i.e., protecting the product from microorganism contamination that may occur during the distribution process and extending the shelf life of the product. The search results showed the dominance of active packaging (53 occurrences), with the addition of antimicrobial (20 occurrences) or antioxidant (17 occurrences) compounds to prevent the oxidative degradation of the packaged food components. Essential oil (Eos) (18 occurrences) derived from plants and spices has antibacterial and antioxidant characteristics. Owing to its strong aroma, the use of EOs as a food preservative is often limited. Thus, to overcome this, EOs can be incorporated into packaging films [61]. Bioactive components rich in antioxidants and antimicrobials such as curcumin show a promising current trend in packaging (Figure 7b). In the food packaging industry, sustainable (6 occurrences) and biodegradable materials are trending, including biopolymers, biocomposites, and bionanocomposites. Biopolymers (24 occurrences) are natural polymers derived from plants and animals, such as polysaccharides (12 occurrences) and proteins (7 occurrences) [62]. Then, biocomposites (6 occurrences) are composite materials consisting of natural polymers and solid particles [15]. Meanwhile, a bionanocomposite (7 occurrences) is a biocomposite with nanoparticles as solid particles [28]. As presented in Figure 7b, biocomposites have been a hot topic of discussion as of late, whereas keywords related to nanocomposites tend to appear in older publications. Biocomposite materials are considered superior to nanocomposites as packaging materials for their renewability, biodegradability, and compostability, thus minimizing waste consumption on the environment.

The third cluster (blue) focuses on film properties. The ability of food packaging to protect food and withstand physical stresses during storage, transportation, and handling is largely determined by its mechanical properties (28 occurrences). Some of the key mechanical properties of food packaging include barrier properties (14 occurrences), water-vapor permeability (11 occurrences), oxygen permeability (5 occurrences), tensile strength (6 occurrences), and antimicrobial (15 occurrences) and antioxidant (23 occurrences) activity as additional properties. The film should serve as a strong barrier to prevent the ingress of oxygen, moisture, and other substances that can degrade the quality and freshness of the packaged food. Tensile strength indicates the amount of tension required to tear a film sample. Films should have a high tensile strength to avoid unwanted tearing. Permeability to gases such as oxygen is also important as it controls fruit ripening and can reduce the oxidation of certain food components such as polyunsaturated fats [63,64]. These properties must be balanced to ensure that the packaging can protect food products and is cost-effective and consumer-friendly.

## 5. Application of Biodegradable Films Packaging for Food

The COVID-19 pandemic has changed people’s lifestyles, especially in choosing food products to consume. Consumers tend to choose products that are natural, safe, hygienic, and packaged with materials that are biodegradable, recyclable, and environmentally friendly [65,66]. Biodegradable packaging has been developed to replace conventional plastics and is an alternative to meet consumer demands. Recently, to extend the shelf life of the packaged product, the use of biodegradable packaging is often associated with the addition of active ingredients such as antimicrobial agents. The application of biodegradable film packaging in food products added with active ingredients has been investigated periodically (Table 2).

Several researchers have reported on the use of plant extracts, EOs, and organic acids as antimicrobial agents in biodegradable active films rich in phenolic compounds [67,68,69,70]. Phenolic compounds can serve as ionophores in lowering the pH gradient across the membrane of microorganisms. Thus, the proton motive force is reduced, which is followed by inhibited nutrient absorption and results in bacterial cell death [71]. The application of natural plant extracts in biodegradable active films was studied by Joanne et al. [69] using durian leaf extract at different concentrations (0.5% and 0.2%) as the active ingredient (polyphenols) to develop gelatin films. The gelatin film with 0.5% durian leaf extract showed 17.6 times higher DPPH scavenging activity than without durian leaf extract (control) and improved the functional properties of the packaging film in slowing down oil oxidation. Verdi et al. [72] also explored antimicrobial agents from *Moringa oleifera* extract (1%, 3%, 5%, and 10%) in the preparation of polybutylene adipate terephthalate (PBAT) films applied to strawberries. PBAT films added with *Moringa oleifera* extract could reduce fungal contamination, and the resulting films showed good thermal stability. PBAT + *Moringa oleifera* 1% film showed good results in strawberry storage packaging. Extracts from curcumin, rosehip, and mango peel also successfully enhanced the antioxidant and antimicrobial potential of biodegradable packaging films [73,74,75].

Recently, the use of plant waste extracts to add and improve the quality of environmentally friendly packaging films in warding off free radicals has been hotly discussed. Accordingly, Kanatt and Chawla [75] explored the potential of mango peel extracts of various varieties in polyvinyl alcohol (PVA), cyclodextrin, and gelatin films. Langra mango peel extract has the highest phenolic content of 235 mg/g. In addition, Langra fruit peel has the lowest IC50 value, which shows the highest DPPH radical scavenging activity and the best antibacterial activity against Gram-positive (*Staphylococcus aureus*) and Gram-negative (*Pseudomonas fluorescens*) bacteria. Another study revealed that PLA-based biodegradable film packaging and natural olive waste extract (20% w/t) efficiently increased antioxidant effects and slowed down the oxidation/browning reaction of freshly cut avocadoes [76].

The antimicrobial and antioxidant properties in food can be also obtained from the use of EO. The antimicrobial properties of EOs are often associated with their active compound components and hydrophobic properties [77]. For example, Kamkar et al. [78] examined the effect of nanoliposomal garlic EO (NLGEO) concentration on the properties of CS biodegradable films applied to chicken fillets. The addition of garlic EOs could improve the mechanical characteristics and water resistance of the resulting film, and the lowest growth of *S. aureus* and coliforms was observed in 2% NLGEO film at 2.98 log cfu/g. Similarly, Dirpan et al. [79] used garlic extract with various concentrations on cellulose-based films. They found that films added with 10–15% garlic extract extended the shelf life of beef up to 4 h (28 ± 2 °C), longer than the control, and provided antimicrobial activity against *S. aureus*.

A study Lee et al. [80] that used thyme EO in skin gelatin-based active films showed an increase in antibacterial activity on *Listeria monocytogenes* and *Escherichia coli* O157:H7. Similarly, Sayadi et al. [81] used cumin EO in alginate/TiO_2_ active films, which reduced the total mesophilic bacteria and lipid oxidation, extending the shelf life of beef. Cardoso et al. [82] and Souza et al. [43], respectively, used *Origanum vulgare* oil (EOE) and cinnamon EO in PBAT-based active films and reported that these oils successfully provided active functions in biodegradable films and improved the elastic modulus, elongation, and thermal stability properties of the resulting films.

Organic acids have the potential to serve as antimicrobial agents in food by ionizing acid molecules, which can lead to alterations in the permeability of bacterial cell membranes. This, in turn, causes damage to the extracellular membrane and disrupts the intracellular pH balance, ultimately resulting in bacterial destruction [83,84]. Moreover, several researchers have examined the role of organic acids in biodegradable films as antimicrobials. Wen et al. [68] examined the antimicrobial potential of citric acid in PVA films. As a result, PVA films added with citric acid were found to provide antibacterial properties and were more sensitive to *S. aureus* than *E. coli* with an inhibition zone area of 12 mm. Similarly, ref. [85] used citric acid and curcumin as antimicrobial agents in CS and chickpea flour (CF) films, which effectively inhibited the growth of *S. aureus* and *E. coli*. Thus, CF films can maintain the shelf life and appearance of chicken for 9 days of storage at 4 °C.

**Table 2:** Application of biopolymer-based biodegradable films packaging.

Type of Polymers	Application	Active/Antioxidant Compound	Characteristic Packaging/ Improved Features	Ref.
CS	Chicken fillet	Garlic essential oil	■ Garlic essential oil enhanced the mechanical characteristics and water resistance of active films. ■ Active films significantly reduced the growth of microbes on refrigerated chicken fillet.	[78]
Cellulose/potato peel (PP)	Fresh pork	Curcumin	■ Bacterial cellulose improved the mechanical characteristics of PP films while decreasing their light transparency, oxygen permeability, and water-vapor permeability. ■ Curcumin provided the film with strong antioxidant activity. ■ PP films successfully reduced the lipid oxidation of fresh pork.	[73]
PLA/PBAT	Bakery	Carvacrol	■ Carvacrol-containing films with concentrations of 2% and 5% inhibited fungal growth and sporulation and extended the shelf life of packaged bread and butter cake to 4 days. ■ Carvacrol-containing films with a concentration of 2% were recommended because of their comparable antifungal properties to those containing 5% carvacrol.	[44]
ZNO/CS	Fresh poultry and minced meat	Zinc oxide nanoparticles	■ The film-protected samples exhibited a reduction in the rates of degradation, oxidation, and microbial growth. ■ The films extended the shelf life of fresh poultry meat.	[86]
Corn starch	Strawberry and ricotta	Chitosan oligomers	■ Sachet-type packages were developed from active films with notable antimicrobial capability against molds and yeasts.	[67]
PBAT/PLA	Shrimps	Carvacrol, citral and α-terpineol	■ The release of carvacrol and citral from films slowed the deterioration of shrimp quality. ■ Films containing 6% citral provided the greatest rate of melanosis inhibition (up to 3 times). ■ Essential oils in films inhibited the growth of microorganisms and the loss of shrimp heads and drips.	[42]
PBAT/thermoplastic starch (TPS)	Fresh noodles	Sorbate and benzoate	■ Films containing 3% sorbate and benzoate reduced the growth of microorganisms on fresh noodles. ■ 6% sorbate produced homogeneous microstructures, enhancing film transparency and permeability.	[87]
Skate skin gelatin (SSG)	Chicken tenderloin	Thyme essential oil	■ Increased antibacterial activity was observed against *Listeria monocytogenes* and *Escherichia coli* O157:H7.	[80]
Purple yam starch (PYS)/CS/glycerol	Apples	Chitosan	■ Four-week application of film on apples maintained the fruit’s quality. ■ Glycerol contributed to the thermal stability of films.	[88]
PVA	Strawberry and cherry tomatoes	Citric acid (CA)/carboxymethyl chitosan (CMCS)	■ Films made of PVA/15 CMCS and PVA/15 CMCS/2.5CA can delay evaporation and water loss. ■ Films significantly reduce the growth of bacteria.	[68]
Gelatin	Fresh durian cut	Durian leaf extract	■ The DPPH scavenging activity of a gelatin film containing 0.5% leaf extract was 17.6 times greater. ■ The use of 0.5% leaf extract in a gelatin-based film was more effective in retarding oil oxidation. ■ The durian leaf extract did not increase the water-vapor permeability of gelatin films.	[69]
Triticale flour	Cheese	Natamycin	■ Natamycin-treated triticale flour films inhibited the formation of mold on the surface of soft cheese.	[89]
Fish skin gelatin (FSG)	Cheese	*Moringa* *oleifera* Lam. leaf extract (ME)	■ The FSG film containing ME demonstrated antioxidant and antibacterial action against *Listeria monocytogenes*. ■ Films effectively inhibited microbial growth and retarded the lipid oxidation of cheese.	[90]
PLA	Beef	Nisin/ε-poly lysine (ε-PL)	■ ε-PL-g-PLA and nisin-g-PLA films successfully inhibited *S. aureus.* ■ The nisin-g-PLA film was better than ε-PL-g-PLA film in terms of its physicochemical and antibacterial characteristics.	[91]
Rice flour/PBAT	Pasta	Potassium sorbate	■ The addition of potassium sorbate (1–5%) affected the mechanical characteristics of films. ■ 1–5% of potassium sorbate in blends of rice flour and PBAT is recommended.	[92]
PVA, cyclodextrin, and gelatin	Chicken meat	Mango peel (MP)	■ Composite films using MP exhibited excellent UV light barriers. ■ MP-containing films showed antioxidant and antibacterial activities. ■ Chicken packed with MP-containing films had a longer shelf life (12 days) than the control (3 days).	[75]
Rye starch	Chicken breast	Rosehip extract (RHE)	■ The films were more flexible and had higher RHE concentrations and better light-barrier qualities. ■ Films were suitable as antioxidant films. ■ Rye starch films can effectively inhibit lipid oxidation.	[74]
Curdlan	Fresh pork	Nanocellulose (NC)	■ A film solution of pH 4.5 added with 5% NC addition showed better mechanical properties and barrier properties. ■ Films had a tensile strength of 38.6 MPa and a 40% elongation at break. ■ Films slowed microbial growth and gave products a shelf life of 12 days.	[93]
*U. pinnatifida* protein (UPP)/gelatin	Smoked chicken breast	Vanillin	■ Vanillin showed antibacterial effects on *E. coli* in UPP/gelatin composite film. ■ The addition of high concentrations of vanillin (0.5%) affects the opacity of films.	[94]
CS/pullulan	Goat meat	Carvacrol	■ Carvacrol enhanced the effectiveness of chitosan/pullulan film in blocking UV light. ■ Carvacrol significantly decreases the water vapor permeability (WVP) of the films. ■ The film exhibited excellent antibacterial activity. ■ The shelf life of goat meat can be extended to >15 days.	[95]
PBAT	Strawberry	*Moringa oleifera* (MO)	■ Films showed good thermal stability and decreased slightly for films with high MO content. ■ MO decreased fungal contamination. ■ PBAT-1% MO films performed well as strawberry storage packaging.	[72]
CS/bacterial cellulose (BC)	Grass carp	Tea polyphenol (TP)	■ TP-containing films were more efficient against *S. areus* than against *E.coli* and improve antioxidant activity. ■ The film with 8% TP concentration was the most optimal, with the highest elongation of break, hydrophobicity, and water-vapor barrier.	[96]
Alginate/TiO_2_	Beef	Cumin essential oil	■ Films reduced the population of total mesophilic bacteria. ■ Films can extend the shelf life of fresh beef by decreasing lipid oxidation and microbiological spoilage and improving color quality and sensory qualities.	[81]
PBAT	Mozzarella cheese	*Origanum vulgare* oil (EOE)	■ The films had increased elastic modulus and elongation. ■ The films presented high antioxidant activity and effective antimicrobial activity (*S. aureus*).	[82]
CS/Chickpea flour (CF)	Chicken breast	Citric acid/Curcumin (CUR)	■ Citric acid significantly changed the permeability and mechanical and thermal characteristics and improved antioxidant activity. ■ The highest concentrations of citric acid in the films showed antibacterial activity against *S. aureus* and *E. coli*. ■ Chicken in packed films is still acceptable at the end of the 9-day storage.	[85]
PBAT/PLA	Strawberry	Cinnamon essential oil (EO)	■ Films showed high thermal stability. ■ Films showed a Fickian diffusion mechanism that could be used in active packaging. ■ Films containing PLA capsules prevented weight loss for 30 days while preserving strawberries.	[43]
PLA	Avocado fresh cut	Natural olive wastewater extract (OWE)	■ Films improve antioxidant activity. ■ Films increased the equilibrium time by increasing the antioxidant concentration.	[76]

## 6. Biodegradable Film as a Current Trend in the Food Sector

Plastics that enter the environment as large or small plastic pieces can cause various environmental problems. They can change the way ecosystems work and harm living things. Eventually, they can end up in the food chain, which can harm human health. At present, stopping plastics from entering the environment in various forms is impossible. Thus, reducing environmental pollution caused by plastics or microplastics becomes more important. Recently, biodegradable film packaging is a good and attractive option for plastics that can degrade in the environment. Biodegradable plastics will decompose completely over time, whereas non-biodegradable plastics remain in the environment for hundreds of years.

**Figure 8:**
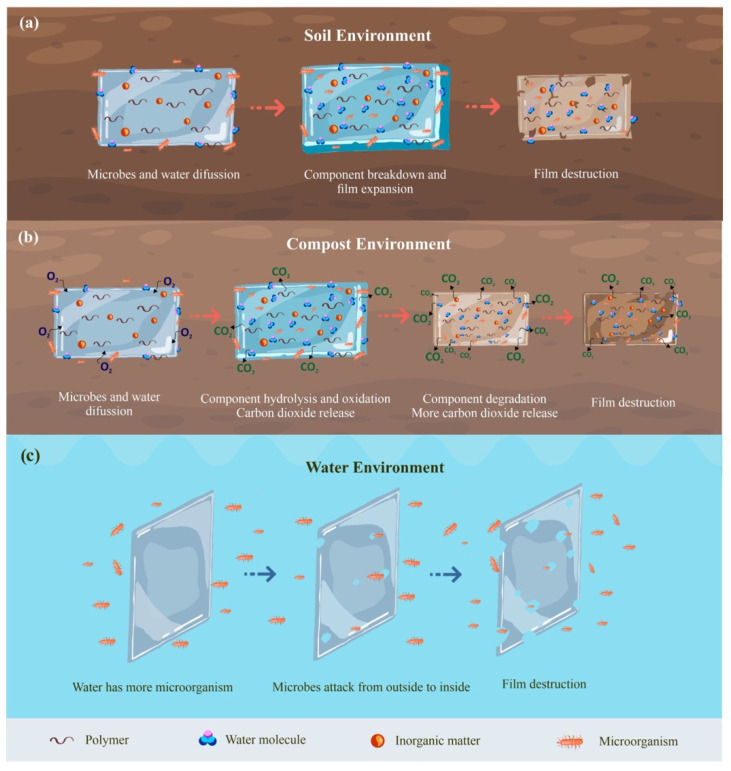
Degradation mechanism and behavior of biopolymer-based composite films at different degradation environments: (**a**) Soil environment; (**b**) Compost environment; (**c**) Water environment.

A plastic is biodegradable if all its organic parts break down into carbon dioxide, water, mineral salts, and biomass under anaerobic conditions or carbon dioxide, methane, mineral salts, and biomass under aerobic conditions. During the biodegradation of plastics, some of the carbon is released into the atmosphere as CO_2_/CH_4_, whereas the rest is used to grow biomass such as microorganisms and fungi. The chemical structure of the polymer and surrounding environmental conditions greatly influence the biodegradation process (Table 3). The biodegradation rate is affected by temperature, amount of water, nutrient availability, pH, amount of oxygen, concentration and activity of microorganisms, etc. Under the same environmental conditions, the decomposition rate of different products or materials may also vary. The biodegradation rate is affected by temperature, amount of water, nutrient availability, pH, amount of oxygen, concentration and activity of microorganisms, etc. Under the same environmental conditions, the decomposition rate of different products or materials may also vary. As a result, it is necessary to take into account the biodegradation characteristics of biopolymer-based films in different environments. The biodegradation process and mechanism of biopolymer-based composite films in different degradation environments are summarized in Figure 8.

### 6.1. Soil Burial

Compared with petroleum-based plastic packaging, polymer packaging materials can biodegrade and decompose under the influence of microorganisms found in the environment. Soil stockpiling can be used to determine important details of the biodegradation process and illustrate the actual state of the biodegraded material. Soil conditions vary widely. Some soils are wetter and have more microorganisms than others. Differences in temperature and pH can also slow down the biodegradation rate. In soil and compost, scientists have found >90 types of microorganisms that can recycle biodegradable plastics. Generally, the film degradation process in soil occurs in two stages, starting with water diffusing into the film causing the film to swell accompanied by the growth of microorganisms, which is followed by secretory degradation induced by enzymes and other substances, resulting in weight loss and film destruction [15].

Zehra et al. [97] revealed that CS/thyme EO blended films combined with ZnO/polyethyleneglycol (PEG)/nano clay (NC)/calcium chloride (CaCl_2_) have a low water vapor transmission rate and high tensile strength, and they are water soluble and biodegradable. CH/TEO films have the highest biodegradation rate for 28 days. Similarly, Mohan et al. [98] found that CS/mustard oil films degraded by 45–70% after 21 days of burial in soil. Yu et al. [99] and Sarojini et al. [100] used CS/polyurethane (PU) and CS/PVA, respectively, and they found that the film degraded 63–80% after 28–30 days of burial in soil. Different results were reported by Bashir et al. [62] who incorporated CS/PVA/guar gum in films, where nearly all films degraded rapidly in only 6 days. The degradation time of films differed because different films have different hygroscopicity, which may cause inaccuracies in weight measurements. In addition, the amount of residues on the film surface will directly affect how much weight is lost over time [101]. The degradation performance of composite films or mixtures is influenced by the film constituent materials and soil properties. Importantly, different locations, seasons, and rainfall have led to different soil qualities, and these elements directly influence how quickly the film can degrade in soil. Moreover, the exchange of gases and liquids in the soil and environment is affected by the particle size of the soil. When the particle size is <2 mm, the soil is thick and has little exchange space with the environment and vice versa [102].

The soil environment contains various microorganisms, such as bacteria and fungi, that can use biopolymers as their energy source and convert them into carbon dioxide, water, and new biomass, which in turn can contribute indirectly to the synthesis of various biopolymers [103]. As an advantage, the use of soil burial to test the biodegradation of composite films or blends provides the most accurate picture of the environment and film deterioration process, and the testing cost is relatively low. Soil burial also has the following disadvantages: (1) it takes a long time, and most experiments take months; (2) biodegradability determined by weight loss sometimes cannot accurately reflect the actual results because removing soil, debris, and attached microorganisms from the material is difficult; and (3) degradation characteristics generally cannot be determined through repeated testing because of regional dependence.

### 6.2. Compost Environment

Composting (also known as organic recycling) is a biodegradation process that occurs under certain circumstances, depending on time, temperature, and the presence of microorganisms. Composting indicates that the material not only decomposes but also contributes nutrients to the soil in addition to being a usable component of the compost [35]. In the composting process, the relative humidity is generally controlled at 40–55%, and the pH value is 6.5–7.5 [104].

Several researchers have conducted degradation tests of packaging films under degradation conditions on compost. Recently, Mohammed et al. [105] reported that alginate composite films extracted directly from *Sargassum natans* seaweed degraded after 14 days under simulated conditions. Within the first week, an increase in deformation and opacity was observed, indicating the start of the hydrolytic breakdown process. This caused the alginate composite film matrix to crystallize and crack. With a different polymer material, Media-Jaramillo et al. [106] used films from cassava starch added with green tea and basil extracts. The film demonstrated significant degradation after 12 days in composting. Similar to Wongphan et al. [107], mixed PBAT films modified by hydroxypropylated starch (HS) and native starch (NS) reached 99% biodegradation on day 8, and PBAT/acetylated starch (AS) and PBAT/octenyl-succinated starch (OS) reached 97% and 98% biodegradation on days 9 and 11, respectively. Based on these research results, composite/mixed films can degrade quickly in days because the rich microflora of the composting soil likely contributes to the acceleration of film degradation [108]. The characteristics of the composite film/mixture also significantly influence the degradation rate of films during composting. For example, the addition of biodegradable components (CS, starch, protein, etc.) in the films can increase the film’s hydrophilicity so that it degrades quickly. Factors such as temperature and humidity in the compost environment also significantly influence the speed of film degradation [109].

### 6.3. Water Environment

Studies on film biodegradation in aquatic environments mainly focus on seawater, freshwater, and river water. Seawater has highly variable temperatures ranging from 30 to −1 °C, is highly saline (34–37 ppt), and has a lower concentration of microorganisms than freshwater. Freshwater can be stagnant (lakes) and moving (rivers) water, and a significant difference from seawater is that the salt content is lower than 1 ppt. Freshwater has a pH range of 6–9, and biodegradation is generally caused by bacteria and fungi [102]. Abdillah and Charles [108] comprehensively studied the biodegradation rate of arrowroot starch (AS)/carrageenan (IC)-based films in seawater and a compostable environment. Their results revealed that the AS 4% + IC 0% and AS 3.5% + IC 0.5% blend films were completely degraded after 42 days in seawater compared with only 7 days in a compost environment, which is relatively fast, because plastic materials degrade slower in the sea than in the soil environment due to less exposure to thermal oxidation. In addition, films biodegrade faster in the soil environment than in water because the water environment has a relatively low temperature and insufficient microbial abundance. The water environment is also strongly influenced by climate, light, and other factors that affect film biodegradability [102].

**Table 3:** Type and characteristic of degradation polymers.

Type of Polymers	Type of Degradation	Degradation Parameters			Degradation Characteristics	Ref.
		Temperatures	Degradation Period (Days)	Test Method		
CS/thyme essential oil (TEO)	Soil burial	NA	28	Weight loss	■ CS–TEO composite film showed a high degradation rate compared with other composites ■ The composite film shows the lowest degradation rate	[97]
Cellulose/carboxymethyl cellulose/*snail mucus* extracted	Soil burial	NA	30	Weight loss	■ After 2 weeks, film with the addition of CMC and snail extract 70% decreased by 54% ■ After 4 weeks, it is completely biodegradable	[110]
PVA	Soil burial	NA	5	Weight loss	■ 40% degraded PVA + glass flakes film ■ 60% degraded PVA film	[111]
Starch/PBAT	Composting	58 °C	18	Percent biodegradation	■ 80% PBAT + TPS film degraded over 5–7 days ■ PBAT/NS and PBAT/HS reached 99% biodegradation on day 8 ■ PBAT/AS and PBAT/OS reached 98% biodegradation on day 11	[107]
Starch/PVA	Soil burial	27 ± 5 °C	21	Weight loss	■ Pure starch degrades completely in 7 days ■ Pure PVA and SP70 completely degraded in 21 days	[112]
Starch/carrageenan	Seawater	NA	70	Visualization (digital camera)	■ High-concentration arrowroot starch (AS) films have the fastest degradation time compared with high iota-carrageenan (IC) films ■ The 4% AS + 0% IC and 3.5% AS + 0.5% IC films were completely degraded after 42 days ■ AS 2% + IC 2% film degraded the slowest among other films	[108]
	Composting	NA	30	Visualization (digital camera)	■ The 4% AS + 0% IC, 3.5% AS + 0.5% IC and 3% AS + 1% IC films were completely degraded after 7 days ■ The AS 2% + IC 2% film (with the highest IC) was completely degraded after 30 days	
Hemicelullose/celullose nanocrystal (CNC)/cellulose nanofibril (CNF)	Soil burial	NA	10	Visualization (digital camera)	■ Hemicellulose film starts to degrade on day 6 ■ Hemicellulose + CNC + CNF started to degrade on day 8	[113]
PVA/starch/pectin	Soil burial	30–37 °C	90	Weight loss	■ 12.86% PVA film degraded on day 90 ■ 47–50% of PVA/starch/pectin blend films degraded on day 30 ■ 67–68% of PVA/starch/pectin blend films degraded on day 90	[114]
PVA/carboxymethyl CS (CMCS)/citric acid (CA) films	Soil burial	NA	48	Visualization (digital camera)	■ PVA/CMCS/2.5CA films degraded to a greater extent than PVA/15CMCS films ■ PVA/15CMCS started to degrade on day 30 ■ PVA/CMCS/2.5CA started to degrade on day 14	[68]
Cellulose/CS/castor oil	Compost	25 °C	20	Visualization (digital camera)	■ Film starts to degrade on day 5 ■ Films with high cellulose concentration were completely degraded on day 10	[115]
PVA/chitin	Soil burial	NA	30	Weight loss	■ 52% PVA + chitin film degraded on day 30 ■ 43% silica-reinforced blended film degraded after 30 days of burial	[116]
Hake protein/gluten/zein	Soil burial	NA	60	Weight loss	■ Hake and gluten both showed 100% degradation in the first 10 days ■ At 60 days, zein films lost 74.5% of their weight	[117]
Polyhydroxyalkanoate (PHA)	Soil burial	23 °C	80	Weight loss	■ After 80 days, PHA film lost 75% of its weight	[118]
CS/polyurethane (PU)	Soil burial	Room temperature	28	Weight loss	■ After 28 days, the CS film showed the highest percentage of weight loss (≤83%) ■ After 28 days, the PU film only degrades 50% ■ Blend of PU and CS films degrades 75–80% in 28 days	[100]
CS/PVA	Soil burial	NA	30	Weight loss	■ PVA/CS films degraded 63% at least 30 days	[99]
CS/PVA/guar gum	Soil burial	NA	7	Weight loss	■ On day 2, the film begins to degrade ■ Almost all films degraded rapidly in 6 days	[62]
Gelatin/dialdehyde xanthan gum (DXG)	Soil burial	Room temperature	30	Weight loss	■ Within 30 days, all edible films biodegraded almost completely (>92%) ■ Gelatin films without crosslinking degraded in 7 days ■ Gelatin-based edible films’ biodegradation can be slowed down by DXG crosslinking	[119]
Gelatin	Soil burial	NA	15	Weight loss	■ After 15 days, all films were reduced by 68% of their initial weight ■ The films lost their original form after soil burial as a result of water absorption	[120]
Starch	Soil burial	NA	15	Weight loss	■ The weight loss of all films ranged from 52.98% to 61.22%	[121]
Starch/glycerol	Compost	NA	12	Visualization (digital camera)	■ Both films rapidly degraded within the first 6 days, with a significant loss of mass ■ After 12 days, films showed significant degradation ■ The use of tea and basil extracts led to shorter degradation times in soil	[106]
Cellulose/*S. urens* short fiber (SUSF)	Compost	30 ± 2 °C	40	Weight loss	■ SUSF/cellulose composite films showed a faster rate of composite degradation ■ The weight decreases of all films averaged 70% over 25 days	[122]
CS/PVA	Soil burial	NA	15	Weight loss	■ The degradation rate of the controlled CS/PVA film shows 26.41% ■ The CPW (solanum water extract) composite film shows higher degradation (50%) than the controlled CS/PVA film	[123]
Gelatin	Soil burial	NA	49	Weight loss	■ Gelatin-based films fully degraded after 7 weeks ■ Films exhibited perfect mechanical strength	[124]

NA: Data not available.

## 7. Commercial Application of Biopolymer-Based Biodegradable Film

Companies operating in the green-packaging industry offer various packaging solutions for various end-user industries. In addition, they offer customized packaging products to meet customer-specific needs [125]. Table 4 provides some of the leading suppliers in the global biofilm packaging market.

Amcor PLC (Zutphen, Netherlands) successfully commercialized cellulose-, starch-, and PLA-based biodegradable packaging films for various food items such as cheese, fresh meat, poultry, fresh fruits and vegetables, and various ready-to-eat foods. The resulting packaging film provides good resistance to heat and has excellent barrier properties to the aroma, high moisture, and oxygen barrier [126]. In addition, Novamont (Italy) contributed to commercializing starch-based biodegradable packaging films. The packaging film can pack both fresh and dried foodstuffs and has good water-vapor breathability [127]. Similarly, Innovia Films (Wingston, UK) commercialized a cellulose-based biodegradable packaging film. The film packaging can be applied to biscuits, coffee, dairy products, snacks, and various beverages [128].

In 2021, the starch-based product segment was the most lucrative for the biodegradable plastics industry, accounting for >40.0% of the total revenue [47]. Throughout the projection, the starch-based segment will maintain its leading position in the market in terms of both value and volume. In addition, plastics made from PLA products are gaining ground in this market at a rapid pace. One of the major factors that influences this industry is the relatively low cost of PLA compared with those of other products. The global PLA market was valued at USD 700 million in 2019 and was estimated to increase to USD 2500 million by 2025 [47,129].

**Table 4:** Commercial application of biodegradable films for food packaging.

Suppliers	Materials	Brand Names	Application	Properties	Ref.
Innovia Films (Wingston, UK)	Cellulose	Propafilm TM RC30	Biscuits, cookies, crackers, bakery	■ Moisture barriers ■ Barriers against oxygen and mineral oils ■ Aroma protection	[128]
		Propafilm TM FFF	Candy and confectionery	■ High-barrier films ■ Cold seal ■ Films with controlled shrinkage	
		Propafilm™ Strata SL	Coffee	■ High-oxygen barrier ■ Aroma barrier ■ Mineral oil barrier	
		RayoForm™ Propafilm™	Dairy products	■ Packages designed for recycling ■ Enhanced shelf appeal	
		Propafilm™ Strata	Granola (nutritional bars)	■ High-barrier films ■ Peelable films ■ Cold seal options	
		Propafilm™ QLD Propafilm™ QID	Ice cream and frozen novelties	■ Packages designed for recycling ■ Enhanced shelf appeal	
		RayoWrap™	Juice and sports drinks	■ Monoweb films ■ No powdering ■ High gloss and shelf appeal	
		Propafilm™ QLD Propafilm™ MPM 17 Propafilm™ GPD 17	Snacks	■ High-barrier films ■ Wide heat seal ranges ■ “Made for Recycling” certified	
		PropafilmTM	Tea and infusions	■ Moisture and aroma barrier ■ Anti-static films ■ Mineral oil barrier	
Amcor (Zutphen, Netherlands)	Cellulose, starch, and PLA	HeatFlex™	Shelf-stable ready meals, juices, smoothies, and sports and energy drinks	■ Excellent barrier properties (aroma) ■ Up to 60% reduced carbon footprint	[126]
		PrimeSeal™	Fresh meat and poultry	■ Films range 70–250 microns in thickness ■ Heat resistance up to 100 °C	
		DairySeal™	Cheese	■ Films range 70–250 microns in thickness ■ Heat resistance up to 100 °C	
		AmPrima™ PE Plus	Dry baby food, milk formula, coffee, cereals, nuts and dried fruits; liquid pouches: yogurt and fresh cheese, juices and smoothies, and other dairy products	■ High-moisture and oxygen barrier ■ Excellent shelf life ■ Outstanding shelf appeal	
		AmPrima™	Fresh fruits and vegetables, frozen fruits and vegetables, cereals, snack bars, cheese, frozen meat, poultry, and coffee	■ Resistant to damage ■ Recycle-ready	
Bio4Pack (Haaksbergen, Netherlands)	Starch and PLA	Bio4Pack	Rice, grain, cookies, perishable products such as meat, and crisps	■ Compostable ■ Aroma and flavor barriers	[130]
Novamont (Italy)	Starch	Mater-Bi	Fresh and dry foodstuffs	■ Good moisture vapor breathability ■ Biodegradable and compostable packaging	[127]
Plantic Technologies Ltd. (Jena, German)	Starch	Plantic™	Meat, snack, coffee, and dairy products	■ Ultrahigh barrier	[131]
Taghleef Industries (Koblenz, German)	PLA	Extendo^®^	Bakery, coffee, snacks, ice cream, and freshly cut produce	■ High- and ultrahigh gas barriers ■ Moisture barrier ■ Aroma and mineral oil barriers	[132]
	Cellulose and PLA	Nativia^®^	Fresh products, bakery, dairy-perishable, snacks, and confectionery	■ Heat sealable and biodegradable	
Sidaplax (Ghent, Belgium)	PLA	Earthfirst^®^ Biopolymer Films	Fresh and dry products	■ Very high yield ■ Flavor, aroma, and grease barrier properties	[133]
Clondalkin group (Wieringerwerf, NL)	PLA	Wentus (Wentopro^®^)	Fresh and dry products	■ Barrier films for food product presentation, protection, and conservation ■ High-performance skin films ■ Compostable films	[134]

## 8. Overview of Social, Environmental, and Economic Aspects

The United Nations has set 17 Sustainable Development Goals (SDGs) that seek to eradicate poverty and hunger, reduce inequality, and advance environmental, health, and education concerns [135]. The use of biodegradable packaging films can directly contribute to realizing the SDGs listed in the 2030 Sustainable Development Agenda.

In this review, the use of packaging films can help achieve SDG 2 (zero hunger), as it can maintain the quality of packaged products in terms of appearance, texture, and taste. Thus, to extend the shelf life and reduce wasted food (food waste), Klinmalai et al. [44] used PLA/PBAT film + carvacrol to extend the shelf life of bread and butter cake packaging up to 4 days of storage at 25 °C. Foodborne diseases caused by pathogenic microorganisms present on food surfaces are emerging as a public health problem worldwide [136]. Thus, the incorporation of substances in packaging films that have antioxidant or antimicrobial activity can have a beneficial effect on achieving SDG 3 (good health and well-being), which aims to ensure food safety and quality. In cellulose films added with curcumin addictive substances [73], curcumin provides strong antioxidant activity and can reduce fat oxidation in fresh pork. Similarly, Sayadi et al. [81] added cumin EO as an antimicrobial agent in alginate/TiO_2_ films and reported that the film can reduce the population of total mesophilic bacteria and extend the shelf life of fresh beef while maintaining its color and sensory quality.

Studies on single-component films’ properties are increasing, which is required for their use and commercialization as innovative food packaging materials. This could lead to “customized” packaging materials for sectors that support SDG 9 (industry, innovation, and infrastructure). As shown in Table 4, this has commercialized various biodegradable packaging films for packaging foodstuffs. One of the suppliers is Bio4Pack (Haaksbergen, Netherlands), which makes packaging films from starch and PLA to package rice, grains, cookies, and perishable products such as meat and crisps [130]. Novamont (Italy) and Plantic Technologies Ltd. (Jena, Germany) commercialize starch-based packaging films for packaging fresh and dry products [127,131].

Currently, approximately 5.25 trillion pieces of macro and micro plastics are present in the ocean, and 46,000 pieces are distributed in every square mile of the ocean, weighing up to 269,000 tons. Every day, approximately 8 million pieces of plastic enter the ocean. In addition, food packaging accounts for approximately 9% of the total amount of plastic waste found in the ocean [137]. Replacing conventional synthetic-based packaging with biopolymer-based biodegradable packaging can positively affect the SDGs 11 (sustainable cities and communities), 13 (climate action), 14 (life below water), and 15 (life on land). Packaging films that have high degradation rates in the environment (soil, compost, and water) have been widely discussed (Table 3). For example, hemicelullose/celullose nanocrystal/cellulose nanofibril blend films began to degrade on day 8 during burial in soil [113]. CS/PVA films also degraded by 63% within 30 days of burial in soil [99]. Alginate films completely degraded after 14 days in compost [105], and starch/carrageenan films completely degraded after 42 days in seawater and only 7 days under composting conditions [108]. The use of biodegradable materials for packaging can minimize wastes sent to landfills, and dependence on non-renewable resources can be reduced, which can directly contribute to the achievement of SDG 12, that is, responsible consumption and production. The use of biodegradable film packaging can also help reduce the amount of plastics in the oceans, which is a major threat to marine life and can reduce the amount of plastic pollution in the terrestrial environment, which can harm wildlife and ecosystems.

Considering the volume of scientific publications, researchers and scientists have developed efforts to obtain biodegradable packaging with better characteristics. This is reflected in the numerous scientific papers published. As an illustration, the number of publications in databases in 2022 increased 87% (7-fold) compared with that in 2013 (10 years ago) (Figure 4). Based on this, biodegradable film packaging can significantly reduce the use of plastic made from non-biodegradable polymers and can reduce wastes. In addition, biodegradable film packaging will better protect packaged food from pathogen contamination, help maintain quality, and can reduce losses due to food loss/waste. Generally, biodegradable packaging films have great potential for use in packaging food products. However, potential challenges must be overcome for large-scale applications, which mainly involve controlling costs to make the process competitive with commercially available packaging systems. The use of biodegradable packaging films can contribute to a more sustainable and environmentally friendly future while advancing progress toward the SDGs.

## 9. Conclusions: Limitation, Challenges, and Future Perspectives

This literature review examines the growth and trends of research on biodegradable films in food packaging using a bibliometric approach and literature review. Based on the analysis, this theme began to be widely discussed in 2019, which peaked in 2022 with 77 articles and 15 reviews. As regards the number and distribution of publications by country, Brazilian and Chinese researchers predominated and are responsible for 20.4% and 12.5% of published papers, respectively. Then, keyword analysis by period showed that the addition of bioactive components rich in antioxidants and antimicrobials, such as curcumin, to packaging films provides a promising trend and is being hotly researched today.

Biodegradable films for food packaging have various benefits; especially, it can positively affect the environment. However, this packaging film also has several limitations and challenges that must be overcome. First, they have high production costs; thus, cost analysis during production must be reviewed so that these biodegradable films can be widely spread in the community. Second, biodegradable films may not always perform as conventional plastic packaging in terms of durability and barrier properties, which can be an important consideration for food packaging; thus, further studies regarding innovations that can improve the properties of the resulting films such as mechanical, physical, and barrier properties are needed. Third, some biodegradable films can only degrade quickly under certain conditions, such as in a composting environment, and they may not degrade in other environments, such as in the ocean or landfills.

Despite these challenges, the interest in the development of biodegradable films for food packaging is increasing and generating promising future prospects. For example, advances in materials science and engineering could result in the development of new biodegradable films with improved properties and more economical costs. Moreover, awareness to reducing plastic wastes is growing, driving the demand for sustainable packaging solutions, including biodegradable films. Finally, increased collaboration between researchers, industry, and government can help overcome these challenges and accelerate the development of biodegradable films for food packaging.

In addition, this study focuses on mapping research related to biodegradable film for food packaging from 2013 to 2022. However, it is important to note that this analysis may not include all research publications on this issue, as we specifically considered publications indexed in Scopus and did not include indexed publications in Web of Science, PubMed, Google Scholar, and other scientific databases. In addition, it is possible that we missed articles that did not use keywords related to biodegradable film for food packaging. This is because some authors may not mention either in the title, abstract, or keywords, which can lead to bias in our analysis.

## Figures and Tables

**Table 1:** Top countries that produced the most documents on biodegradable films for food packaging.

Rank	Country	Number of Documents	Number of Citations	Total Link Strength
1	Brazil	82	1422	19
2	China	50	1378	19
3	Iran	44	2234	18
4	India	42	908	15
5	Malaysia	30	1217	14
6	Spain	26	2044	21
7	United States	19	884	22
8	Italy	18	177	6
9	Mexico	18	1081	5
10	South Korea	18	448	3

## Data Availability

Available data are presented in the manuscript.
